# Neck schwannoma with phrenic nerve involvement: A rare case report

**DOI:** 10.1016/j.ijscr.2024.109891

**Published:** 2024-06-08

**Authors:** Fatemeh Jahanshahi, Delaram Naderi, Mahboobe Asadi, Maryam Parvizi

**Affiliations:** a Research Committee Member, Faculty of Medicine, Iran University of Medical Sciences, Tehran, Iran; b Otolaryngology Department, Taleghani Hospital, Shahid Beheshti University of Medical Sciences, Tehran, Iran; c Department of Pathology, School of Medicine, Shahid Beheshti University of Medical Sciences, Tehran, Iran

**Keywords:** Neck mass, Schwannoma, Phrenic schwannoma, Neck schwannoma

## Abstract

**Introduction and importance:**

Phrenic nerve schwannoma is an occasional axonal tumor that is mostly asymptomatic.

**Case presentation:**

In this report, a man with a painless lump in his neck was the subject. His diagnostic process included the recording of schwannoma. Phrenic schwannoma was removed by surgery without any complication during follow-up.

**Clinical discussion:**

Surgical excision under general anesthesia was done for the patient and during the surgical explore, the surgeon observed that, the schwannoma arose from the cervical phrenic nerve. The cervical mass was dissected from the phrenic nerve precisely by intracapsular enucleation technique.

**Conclusion:**

The phrenic involvements of schwannomas are extremely rare and mostly presented as a painless mass. Additionally, complete surgical excision of them is an efficient method.

## Highlights

• Phrenic nerve schwannoma is a rare axonal tumor that is asymptomatic in most cases.
• The surgical excision of the phrenic nerve schwannoma is an efficient method for treatment.
• Phrenic nerve schwannoma should consider in differential diagnosis of patients with neck painless mass.

## 1 Introduction

Scarce neurogenic tumors such as schwannomas, which originate from the axonal components of nerves, represent a significant cause of mediastinal masses, accounting for approximately 20–30 % of these tumors [1,2]. This tumor, encapsulated and deriving from Schwann cells, is also known as neurilemmoma [3]. Predominantly observed in males during adulthood, these tumors are often asymptomatic and incidentally diagnosed by physicians. Cases featuring slowly expanding masses or episodes of hiccups have been documented. Schwannomas are associated with spinal ganglia, intercostal nerves, and both parasympathetic and sympathetic nerves [4]. Involvement of the phrenic nerve is uncommon, with sporadic reports in the literature [[2], [3], [4], [5], [6]]. Due to the lack of established guidelines, we present this case to broaden the global understanding of its management. This vignette highlights a middle-aged man with a painless, swollen mass on his neck, diagnosed as phrenic schwannoma through fine needle aspiration cytology (FNAC) and subsequent surgical intervention, without any significant complications. Our report adheres to the SCARE criteria [7].

## 2 Case presentation

**Fig. 1:**
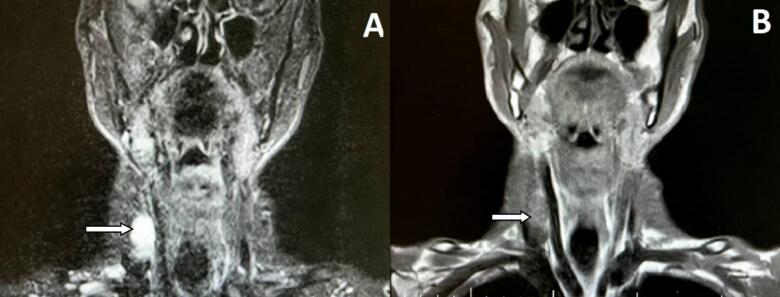
Magnetic Resonance Imaging of the neck, A) a hyperintense and hetrogenous mass on T2-weighted B) a regular Isointense mass on T1-weighted.

A 52-year-old male was referred to the otolaryngology clinic due to a persistent, painless swelling on the right side of his neck, present for the past 10 months. Upon clinical examination, a solitary mass was identified on the right cervical region at level 3, measuring approximately 2.5 × 2 cm. This mass was mobile, non-tender, and exhibited overlying skin that appeared normal. Fine needle aspiration cytology indicated a probable schwannoma. Fiber optic laryngoscopy yielded normal results. Magnetic resonance imaging (MRI) of the neck, performed with and without gadolinium enhancement, revealed a heterogeneous, enhancing mass located on the right side of the neck, with dimensions of 16 × 16 × 23 mm (Fig. 1).

**Fig. 2:**
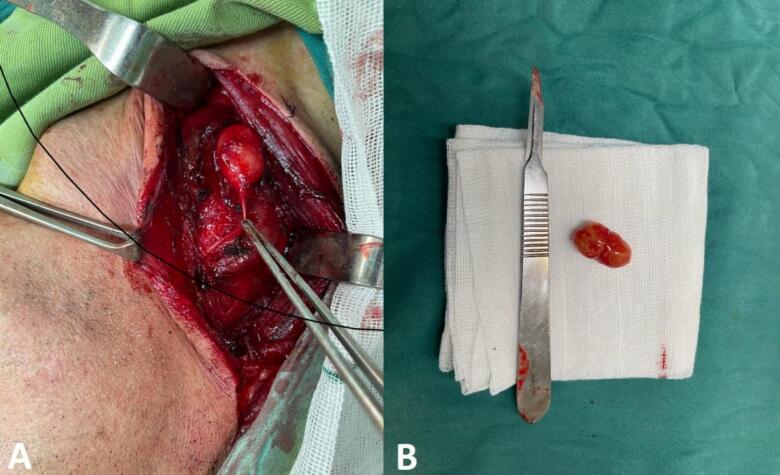
Intraoperative surgical mass resection and gross pathology of the mass.

**Fig. 3:**
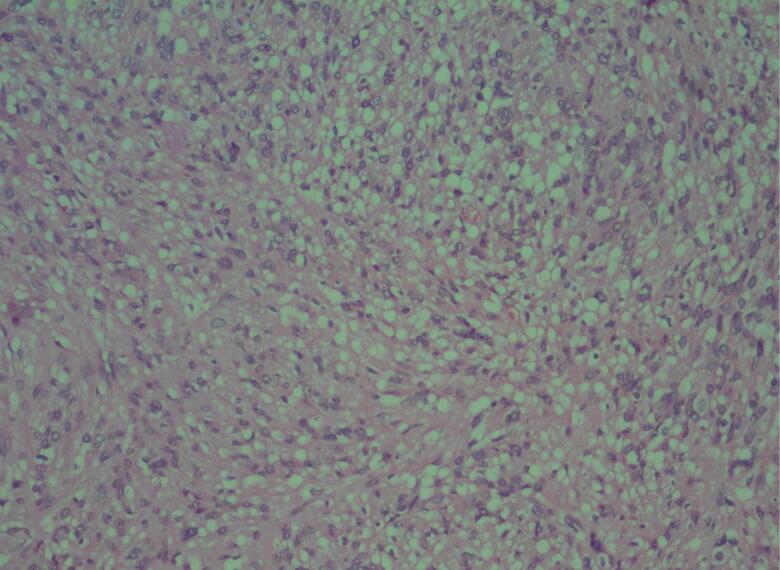
Sections show biphasic neoplasm revealing hypercellularAntoni A and HypocellularAntoni B areas containing bland spindle cells with nuclear palisading (Verocay body).

**Fig. 4:**
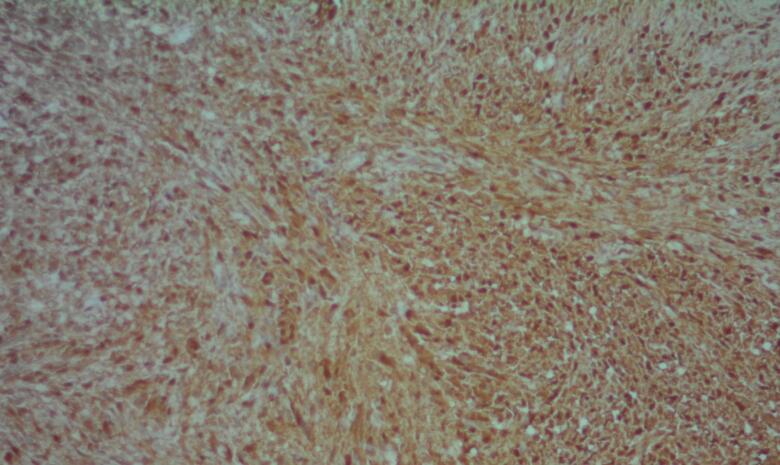
IHC study showed strong nuclear and cytoplasmic immunoreactivity for S100.

The patient underwent a surgical excision under general anesthesia. During the procedure, it was determined that the schwannoma originated from the cervical phrenic nerve. The mass was meticulously dissected from the phrenic nerve through an intracapsular enucleation technique (Fig. 2). The surgical intervention proceeded without significant intraoperative or postoperative complications. Histopathological examination confirmed an encapsulated tumor comprised of spindle cells, consistent with schwannoma (Fig. 3). Immunohistochemistry testing for S-100 protein yielded positive results (Fig. 4).

## 3 Discussion

The primary sources of lesions known as nerve sheath tumors are the cellular structures surrounding the axons of peripheral nerves. These lesions are associated with a deficiency of merlin or schwannomin, which, in their normal state, inhibit cell growth by activating the RAS protein [6]. Schwannomas, typically benign and slow-growing at about 3 mm per year, are solitary, encapsulated lesions with a malignant transition rate of <2 % [[6], [7], [8], [9]]. Intrathoracic neurogenic masses generally located in the posterior mediastinum, originating from either the spinal nerve roots or the intercostal nerves, though they occasionally derive from the nerve sheaths in the parenchymal or intrabronchial, middle, or anterior mediastinal regions. Approximately 25–45 % of extracranial schwannomas occur in the head and neck, primarily in the parapharyngeal space (PPS), with over two-thirds located outside the skull. Schwannomas originating from the phrenic nerve are quite rare [[2], [3], [4], [5], [6]].

In an examination of 138 mediastinal lesions, Tanaka et al. discovered that only one arose from the phrenic nerve [2,9].

These tumors predominantly present without clinical symptoms. They occur more frequently in men between the ages of 30 and 60 and may be linked to neurofibromatosis type 2, Carney complex, schwannomatosis, and other related disorders [2,8].

On the other hand, a considerable body of literature has recorded clinical cases exhibiting specific symptoms, including diaphragmatic paralysis and eventration, persistent dry cough, obstruction of airways and blood vessels, pneumonia, hiccups, and superior vena cava syndrome [3,5,10].

Both non-painful and painful masses, such as chest pain and cervical mass pain, have been documented as well [4,6].

We reported a case involving a 56-year-old male who presented with a solitary, painless, slow-growing lump consistent with characteristics described in the existing literature [3,6,8].

Contrary to the few studies that note rare clinical manifestations, our patient only exhibited a swollen mass on his neck.

Macroscopically, a schwannoma presents as a well-defined mass, ranging in color from gray to pinkish-white or pale yellow, typically of small size (<5 cm), enclosed within a capsule primarily composed of epineurium. Gross characteristics include calcification and degenerative changes. Microscopically, a schwannoma consists of spindle cells organized into hypercellular and hypocellular regions, commonly referred to as Antoni A and Antoni B regions, respectively. Antoni A areas exhibit nuclear palisading, dense chromatin, cytoplasm often arranged in short, crossing fascicles, along with meningioma-like whorls and Verocay bodies. Antoni B regions, on the other hand, are less cellular, characterized by a cellular meshwork with myxoid alterations. Immunohistochemically, S-100 protein is detectable in schwannoma, particularly in Antoni A regions. Notable differential diagnoses include neurofibroma, perineurioma, solitary fibrous tumor, malignant peripheral nerve sheath tumor, and ganglioneuroma. Neurofibroma histologically features oval and elongated nuclei without nucleoli, with scant cytoplasm containing bland-looking spindle cells.

“Perineurioma displays an onion bulb pattern characterized by thin cells and wavy nuclei. Solitary fibrous tumors consist of neoplastic spindle cells interwoven with ‘ropey’ collagen. Malignant peripheral nerve sheath tumors present spindle cells arranged in a fascicular pattern, accompanied by varying degrees of mitosis, necrosis, and tumor calcification when examined under the microscope. Ganglioneuroma stands out from other lesions primarily due to the presence of well-formed ganglion cells.

Our case exhibited an Antoni A appearance with positive staining for S-100 on immunohistochemical analysis [3,6,8].

Some scholars advocate for the routine utilization of fine needle aspiration cytology (FNAC) to discern the etiology of all neck tumors. Nevertheless, the efficacy of FNAC in preoperative diagnosis of head and neck schwannoma remains contentious. The accuracy of FNAC's preoperative diagnosis hinges on both the quality of the sample and the experience of the cytopathologist.” [11].

“Cytological analysis lacks specificity and frequently leads to erroneous diagnoses. The accuracy rate of diagnosing schwannoma through ultrasound-guided FNAC varies widely, ranging from 0 % to 34 % among patients. In gastric cases, FNAC demonstrates an accuracy of 62–93 % in diagnosing subepithelial lesions such as schwannoma [12].

We conducted FNAC for our patient, and the specimen strongly suggested schwannoma. Therefore, we consider FNAC as the primary diagnostic choice. Additionally, neck MRI serves as the gold standard for evaluating phrenic nerve schwannoma and its extension [9].

According to Watanabe et al., if the tumor is related to the interbody between the second and fifth cervical vertebrae, exploration of potential sources of the phrenic nerve or cervical plexus is warranted. As our patient not only presented with a noticeable neck mass but also required differential diagnosis, including infection, and lymphoma, MRI plays a pivotal role in both diagnosis and treatment planning. Our patient's MRI revealed a hypointense signal on T1-weighted images and a hyperintense signal on T2-weighted images.

We conducted Fiber Optic Laryngoscopy, to role out tumors with other neck nerves origine such as vagus and recurrent laryngeal nerves, which present with local cord paralysis.”

“Surgical intervention has been recommended for these lesions in previous studies [[2], [3], [4], [5], [6]]. However, some clinicians advocate for preservation due to their low malignancy rate (0.8). The preferred treatment for cervical schwannoma involves intracapsular enucleation with nerve-sparing techniques. In special cases where nerve preservation is not possible, end-to-end anastomosis or nerve grafting may be required. While “wait and watch” could be considered for benign, slow-growing asymptomatic tumors, they eventually encroach upon surrounding structures, making surgery preferable, especially in younger patients. Schwannomas necessitate surgical excision due to their radioresistant nature and slow growth. Conservative therapy is favored for these tumors, enabling removal without compromising functional integrity. Avascular tumors can be dissected within the capsule after being severed from the parent nerve [[13], [14], [15]].

Regarding follow-up duration, for example, one year could be considered, as no particular complications occurred.

The extent of tumor invasion, size, rapid progression, mass effect, and neurological deficits determine the level of anatomical and/or functional preservation [6,8,9,13]. Ideally, one should tailor the approach based on these clinical parameters. Therefore, intracapsular enucleation was performed in our case due to its size and progressive nature, aiming to prevent mass effect. Regular follow-up about one year revealed no recurrence or complications.”

## 4 Conclusion

Although phrenic nerve schwannomas are exceedingly uncommon and typically manifest as painless masses, they should be considered as a differential diagnosis in similar cases. Recognizing the possibility of this rare condition can facilitate timely and appropriate diagnostic evaluation and management.

## Consent for publication

Written informed consent was obtained from the patient for publication of this case report and the accompanying images. A copy of the written consent is available for review by the editor-in-chief of this journal.

## Ethical approval

Ethical approval for this study was approved by the Ethical Committee of Shahid Beheshti University of Medical Sciences, Tehran, Iran, on February 2022. The present study complies with ethical and research standards involving humans. This article does not contain any studies involving animals performed by any of the authors.

## Funding

This study has no financial source and support.

## Author contribution

Study concept and design: MA.

Acquisition of data: FJ, DN, MP.

Drafting of the manuscript: FJ, DN, MA.

Critical revision of the manuscript for important intellectual content: DN, FJ.

Study supervision: MA.

All authors read and approved the final manuscript.

## Guarantor

Please address all correspondence concerning this manuscript to me at mahboobeh_farvardin@yahoo.com.

## Research registration number

N/A.

## Conflict of interest statement

There is no conflict of interest to declare.

## Data Availability

Data in the current study are available from the corresponding author on reasonable request.
